# The Associations of *Leishmania major* and *Leishmania tropica* Aspects by Focusing Their Morphological and Molecular Features on Clinical Appearances in Khuzestan Province, Iran

**DOI:** 10.1155/2014/913510

**Published:** 2014-09-16

**Authors:** Adel Spotin, Soheila Rouhani, Parviz Parvizi

**Affiliations:** ^1^Parasitology Department, Medical Faculty, Shahid Beheshti University of Medical Sciences, Tehran, Iran; ^2^Molecular Systematics Laboratory, Parasitology Department, Pasteur Institute of Iran, 69 Pasteur Avenue, 1316943551 Tehran, Iran; ^3^Parasitology Department, Medical Faculty, Tabriz University of Medical Sciences, Tabriz, Iran

## Abstract

Cutaneous leishmaniasis has various phenotypic aspects consisting of polymorphic amastigotes with different genetic ranges. Samples were collected from suspected patients of Khuzestan province. Prepared smears were stained, scaled, and measured using ocular micrometer. The Cyt* b*, ITS-rDNA, and microsatellite genes of* Leishmania* were amplified and* Leishmania *species were identified by molecular analyses. Of 150 examined suspected patients, 102 were identified to* Leishmania *species (90* L. major*, nine* L. tropica*, and three unidentified). The amastigotes of 90* L. major* had regular and different irregular shapes within three clinical lesions with no and/or low genetic diversity. Three haplotypes of Cyt* b* of* L. major* were found but no variation was observed using ITS-rDNA gene. Interesting findings were that all nine* L. tropica* had regular amastigote shapes with more genetic variations, also a patient which had coinfection of * L. major*,* L. tropica*, and* Crithidia.* At least two* L. major* and* L. tropica* were identified in suspected patients of the regions. Different irregular amastigotes' shapes of* L. major* can be explained by various reservoir hosts and vectors. In contrast, more molecular variations in* L. tropica* could be justified by genetic characters. Unidentified* Leishmania* could be mixed pathogens or nonpathogens with mammals'* Leishmania* or* Crithidia*.

## 1. Introduction

Cutaneous leishmaniasis (CL) is caused by parasites belonging to the genus* Leishmania* parasitic and referred to neglected tropical diseases [1, 2]. CL is distributed in more than half of the Iranian provinces (20 out of 32) with different prevalence (1.8%–37.9%), also, the number of reported CL cases/year and estimated annual CL incidence are 26,630 and 69,000 to 113,300, respectively [3, 4].

In order of epidemiological importance, three well known principle ethioparasitological agents,* Leishmania (Leishmania) major*; Yakimoff and Schokhor, 1914*, L. (L.) tropica*; Wright, 1903, and less frequently* L. (L.) infantum*; Nicolle, 1908, unequivocally caused CL in Iran [5, 6]. However, recently nonpathogenic* L. (L.) turanica* was reported from human with ZCL (zoonotic cutaneous leishmaniasis) in Iran but authors did not judge that as a causative agent of CL [7].

Different clinical aspects are caused by CL and are observed in several parts of the world wide but many influencing factors of CL have not absolutely understood [8, 9]. The spectrum of genetic diversity in the genus* Leishmania* is described as one of the most controversial issues [10–16]. The genetic heterogeneity/homogeneity of protozoan parasite may bring diverse phenotypic ranges including clinical manifestations or even diversity of morphometric characters in Trypanosomatidae [12, 17–19].

Currently,* Leishmania* species are unambiguously circulating in Khuzestan province, where it is a neglected focus of leishmaniasis in southwestern Iran, coborder with Iraq. The different* Leishmania* parasites' aspects have not been precisely elucidated yet such as ultrataxonomy (strain/haplotype), phenotypic features, and their phylogenetic inferences in Khuzestan province [20–23]. The complexities of these* Leishmania* criteria are associated with the intricacy of leishmaniasis epizoology, migration of people to Iraq and vice versa, recent reactivation of old foci, lack of using accurate diagnostic markers, developing geographical distribution with high temperature (50°C) and humidity (95%), long distance, remote distance from the equipped laboratory, and unsafe sampling in Iraq border [21].

To assess genetic diversity analysis, a number of nuclear and extranuclear DNA markers were employed, including mini-exon, 18S-rRNA, gp63 gene locus, microsatellites, HSP-70, ribosomal internal transcribed spacer regions (ITS-rDNA), minicircles of kinetoplast DNA (kDNA), and cytochrome* b* (Cyt* b*) (kDNA maxicircle) [24–32].

Cyt *b* is the central redox catalytic subunit of quinol which belongs to the mitochondrial genome and it is valuable in understanding phylogenetic study among individuals [33–35].

The ITS-rDNA marker is utilized to infer evolutionary relationships in genus* Leishmania* because of having conserved regions and low intracellular polymorphism [30, 36].

Microsatellites are short tandemly repeated DNA sequence motifs which are distributed plentifully in* Leishmania* species. Microsatellites have a high specificity in identification of* Leishmania* infections owing to their allelic characters [37].

Molecular identification of* Leishmania* species has been conducted by targeting different DNA markers in Iran [29, 38–40]. But in ignored ZCL foci of Khuzestan province, few extensive studies were done on epidemiological and molecular aspects on morphological features of leishmaniases [20–23]. In this research, epidemiological, molecular, and morphological methods were combined with more details on morphological characters of* Leishmania* amastigote shapes, clinical appearances of lesions, and the type of ulcers using nuclear (ITS-rDNA) and mitochondrial (Cyt* b*) genes. The finding of prevalence of CL, molecular variations, and different morphological shapes of* Leishmania* parasite can height our knowledge about behavioral aspects of* L. major* and* L. tropica* in southwestern Iran.

## 2. Materials and Methods

### 2.1. Sampling and Identifying of* Leishmania* Parasites

**Figure 1:**
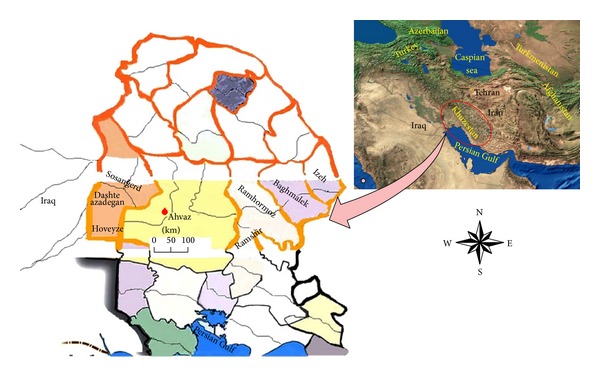
Sampled regions from suspected patients in foci of cutaneous leishmanisis along with their neighboring villages in three geographical locations of Khuzestan province, Iran (western, Dashte azadegan, Sosangerd, and Hoveyze; central, Ahvaz and eastern, Ramhormoz and Ramshir).

In southwest of Iran, within the ZCL foci, three geographical locations, in west (Dashte azadegan, Sosangerd and Hoveyze), central (Ahvaz), and east (Ramhormoz and Ramshir) along with their neighboring villages were sampled (31°7′N to 32°5′N and 47°41′E to 48°30′E) (Figure 1). Some surveyed locations are situated in the border of Iran and Iraq within about 10,406 km^2^ with 12–210 meters above sea level (A.S.L.). The environmental and climate conditions of the studied regions are different from other CL foci in Iran with temperature range: 20–60°C, humidity: 50–85%, and monthly average rainfall: 17–25 mm (Figure 1, Table 1).

The lesions of suspected patients to CL were sampled in 2012-2013. The smears were obtained and prepared in two methods of passive and active for* Leishmania* parasites. The personal information was recorded for each suspected patient (sex, age, lesion duration, type and number of lesion, patients' travelling to endemic regions of ZCL, and drug consumption) (Table 1) [7].

The samples were smeared on two microscopic slides, dried, and stained by Giemsa and* Leishmania* infections were identified under light microscope with high magnification (×1000). The positive slides of each patient were graded to order leishman bodies' density from +1 to +6 [41]. Various morphometric shapes and the sizes of amastigotes of* Leishmania* species were accurately assessed (30 min. per slide) by ocular micrometer (×100 objective = 1 *μ*m per unit space) and Dino Capture 2.0.

Serous of some samples were subcutaneously inoculated into the base tail of the BALB/c and then was examined weekly with the intent of inspecting the appearance of lesion at the position of injection for 6 months. Some serous from active lesion of suspected patients were cultured in Novy-MacNeal-Nicolle (NNN) medium and incubated in 22°C for 6 weeks. The samples were subcultured weekly and checked regularly to monitor the growth and the presence of promastigotes. All experiments on the humans and animals were performed according to the guidelines of the Ethical Board of Pasteur Institute of Iran.

### 2.2. Extraction of Total Genomic DNA

DNA of parasites was directly extracted from graded slides based on modified Phenol-chloroform protocol [42]. All Giemsa-stained slides were washed with ethanol and covered with 300 *μ*L lysis buffer (50 mM NaCl, 50 mM Tris, 10 mM EDTA, pH 7.4, 1% v/v Triton x-100). Giemsa-stained slides should be free from blood in order to prevent misdiagnosis of visualized amastigotes from platelets as well as adverse effects of protoporphyrin on amplified DNA by PCR. The extraction of the genomic DNA of each Giemsa-stained slide was followed by the protocol of Bordbar and Parvizi [29]. DNA was resuspended in 50 *μ*L 1X TE and stored at −20°C. The DNA concentration of each extraction was measured using a NanoDrop (Thermo Scientific Inc., Wilmington, DE). DNA extraction of* Leishmania* parasites was carried out in a molecular biology laboratory where amplified and cloned DNAs had never been performed [30].

### 2.3. PCR Amplification and RLFP for ITS-rDNA, Cyt* b*, and microsatellite Genes of* Leishmania* Parasites from Suspected Patients

The standard PCR was employed to detect* Leishmania* parasites in suspected patients by targeting three genes, ITS-rDNA about 480 bp, Cyt* b* about 880 bp, and microsatellite about 180 bp. PCR products were subjected to electrophoresis in 1.5% agarose gel and were observed under ultraviolet light after staining for 15 min with (0.5 g/mL) ethidium bromide.

**Figure 2:**
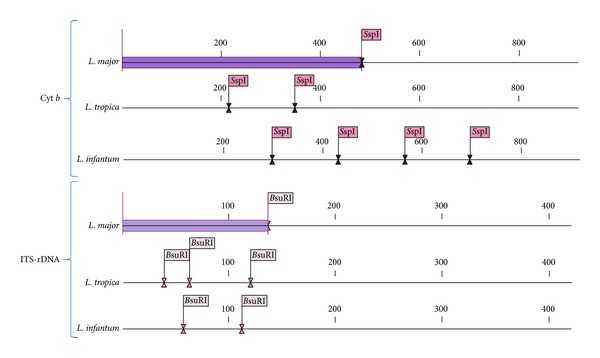
*In-silico* prediction of* S*spI and* B*suR1 restriction enzymes for Cyt *b* and ITS-rDNA genes in the* Leishmania* species analyzed in this study.

**Figure 3:**
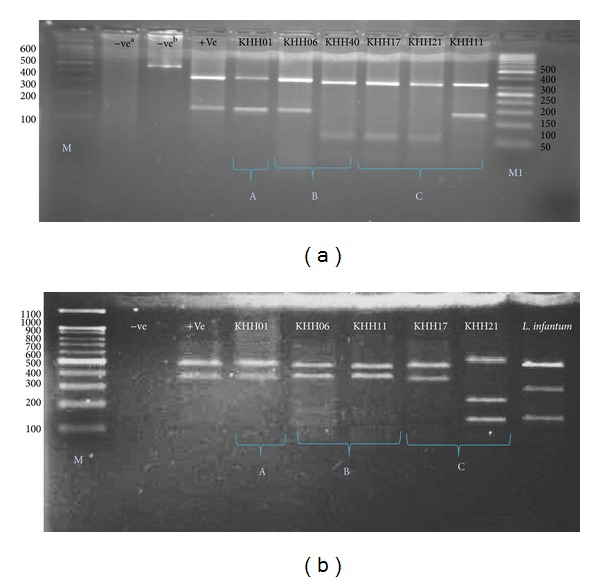
PCR-RFLP observations in Khuzestan isolates based on ITS-rDNA (a) and Cyt* b* (b) genes. A; Lane 1 (−ve^a^: negative control containing* B*suR1 without PCR product), lane 2 (−ve^b^: negative control containing PCR product without* B*suR1), lane 3 (+ve: positive control for* L. major*), lanes 4, 5; KHH 01 and 06* (L. major*): isolated from Eastern and central Khuzestan, respectively. Lanes 6–8; KHH 40, KHH 17, and KHH 21* (L. tropica)*: isolated from central and western Khuzestan, lane 9; KHH 11;* L. major* isolated from western Khuzestan. M: 100 bp size marker. M1: 50 bp size marker. −ve: negative and +ve: positive. B; lane 1 (−ve: negative control containing* B*suR1 without PCR product), lane 2 (+ve: positive control for* L. major*), lanes 3–6; KHH 01 and 06 KHH 11 and 17* (L. major*): isolated from eastern and central and western Khuzestan, respectively. Lane 7; KHH 21* (L. tropica*): isolated from western Khuzestan, lane 8;* Leishmania infantum* with reference strain; MHOM/TN/80/IPT1. A; isolated samples from eastern Khuzestan, B; isolated samples from central Khuzestan and C; isolated samples from western Khuzestan.

For digesting the PCR amplicons in RFLP, the suitable enzymes were selected. The sequences of ITS-rDNA and Cyt* b* genes of Old World* Leishmania* were retrieved (ITS-rDNA:* L. major*: EF413078,* L. tropica*: KC540906, and* L. infantum:* EU330402; Cyt * b*:* L. major:* AB095961,* L. tropica*: EF579904, and* L. infantum*: EF579895). To standardize the number and the size of DNA fragments for each* Leishmania* species, the retrieved sequences were designed by* in-silico* analysis software CLC DNA Workbench 5.2 (CLC bio A/S, Aarhus, Denmark) (Figure 2). Endonuclease reaction of ITS-rDNA and Cyt* b* genes was performed in a volume of 30 *μ*L containing 2 *μ*L of* B*suRI (*H*aeIII) with cut site GG↓CC and 2 *μ*L of* S*sp1 with cut site AAT↓ATT, 10 *μ*L of PCR products, 2 *μ*L of 10x buffer, and 16 *μ*L of distilled water for 4 h (delayed digestion) at 37°C for ITS-rDNA gene and 5 min. (rapid digestion) at 37°C for Cyt* b* gene. Standard strains of WHO for* L. major* (HOM/SU/73/5ASKH),* L. infantum* (MHOM/TN/80/IPT1), and* L. tropica* (MHOM/PS/2008/344Jn SF53) were used as positive controls. The negative controls were applied, one of which had no PCR product and the other one had no restriction enzyme. After digesting PCR products, the fragments were analyzed using electrophoresis on agarose gel 3% containing ethidium bromide and ladder of 50 bp (Fermentas) (Figure 3).

### 2.4. Evaluation of PCR Band Sensitivities in Three Implemented Genes: ITS-rDNA, Microsatellite, and Cyt* b*

In order to analyze and compare the sensitivity of PCR bands for three genes of ITS-rDNA, microsatellite, and Cyt* b,* the serial dilutions of target DNAs were established in order of 1 : 1, 1 : 2, 1 : 4, 1 : 8, and 1 : 16 for each mentioned gene, afterward, the PCR band intensities of each gene were distinctly calculated in different dilutions based on their chromatogram length using Gel Analyzer software version 3.0.

### 2.5. DNA Sequencing and Phylogeny Analyzing ITS-rDNA and Cyt* b* Genes

To reconfirm the results of RFLP, some amplified PCR products were directly sequenced by targeting ITS-rDNA and Cyt* b* genes in both directions using the ITS1F, ITS2R4 and LCBF1, LCBR2 primers by ABIPRISMTM 3130 Genetic Analyzer automated sequencer (Applied Biosystem, USA). Individual sequences were aligned, justified, and edited in consensus positions compared to GenBank sequences of all regional species in case of homology and congruency of new haplotypes with the use of Sequencher v.4.1.4 Software for PC (Gene Codes Corporation).

Maximum likelihood (ML) tree was constructed via MEGA v5.05 for showing the phylogenetic position of common and new haplotypes of the ITS-rDNA and Cyt* b* sequences based on the Kimura 2-parameter model of nucleotide substitution search by stepwise addition of 100 random replicates and bootstrap values with 1000 replicates [43].

## 3. Results

### 3.1. Morphometric Characterization of* Leishmania* Species and Their Epidemiological Features in Suspected Patients

In total, 150 suspected patients were examined microscopically for* Leishmania* infections among 81,000 population in three different study sites with acute and/or chronic lesions' signs. 137 out of 150 samples (91.3%) were observed microscopically positive with* Leishmania* parasites and cultured in NNN medium (Table 1).

**Figure 4:**
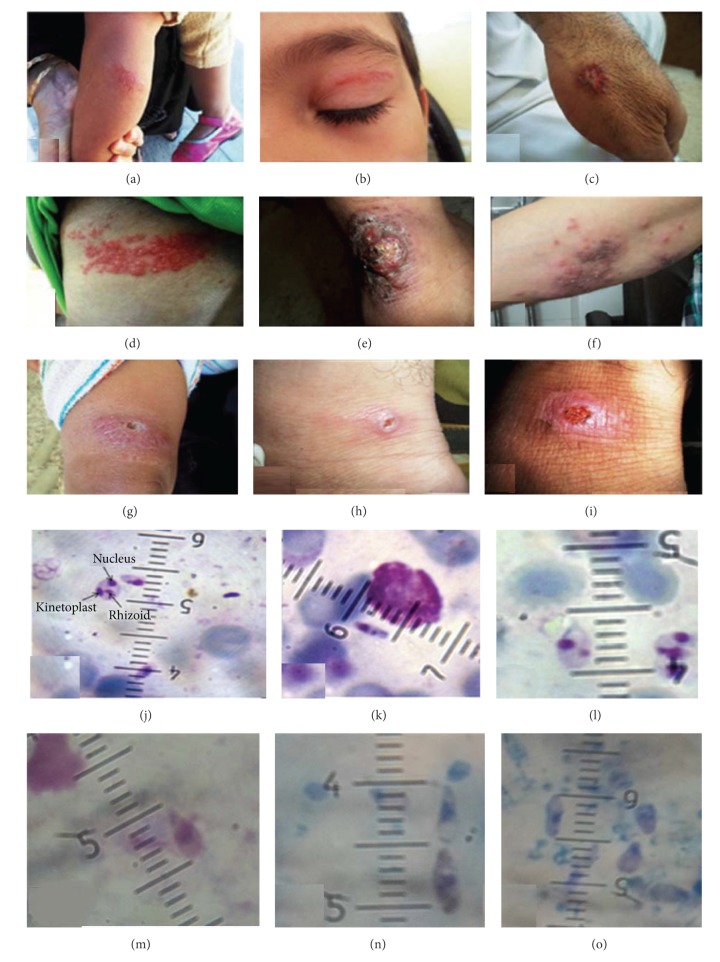
Clinical manifestations of cutaneous leishmaniasis cases in Khuzestan. (a) Dry lesion: isolated from Ahvaz, (b) Dry lesion: isolated from Sosangerd, (c) Mixed lesion: isolated from kotabdollah, Ahvaz, (d) Nonclassical wet-type: Erythematous papulonodules lesion isolated from Ahvaz, (e) Nonclassical wet-type: hyperkeratotic lesion isolated from Ahvaz, (f) Nonclassical wet-type: herpetiform lesion isolated from Hoveyze, ((g)–(i)) classical wet-type form: volcanic lesion isolated from Sosangerd, Ahvaz and Ramhormoz, respectively. (j) The amastigote of* Leishmania tropica* with size 2-3 *μ*m. ((k)–(o)) Polymorphic amastigotes of* Leishmania major* with size 3–5 *μ*m. (k) Cigarette form with size 3-4 *μ*m, ((l)–(o)) pear shapes with size 5 *μ*m.

*Leishmania* infections were found more in 15–25 years old (37/137: 27%) than other groups and in males (94/137: 68.6%). The grades of positive smears were arranged from +1 to +6 (WHO 1991). More infected patients with* L. major* had grade +4 (45/137: 32.8%). The average size of* L. major* amastigotes was in range 3–5 ± 0.31 *μ*m in length and 2.15 ± 0.21 *μ*m in width (Table 2).* L. major* amastigotes were observed with obvious vacuole and polymorphic shapes (regular, oval, and round; 74/90: 82.2%) (Table 2). The irregular polymorphic shapes of* L. major* amastigotes were classified into Cigarette, Spindle, and Pear; 16/90: 17.7% and visualized in three lesion types of wet, dry, and mixed. Interestingly, the sizes of regular and irregular amastigote shapes were approximately 3-4 and 4-5 *μ*m, respectively (Figures 4(k)–4(o)). It is noteworthy that polymorphic amastigote shapes were observed only in one lesion form. The wet lesions were grouped in classic (volcanic sign: 56/70: 80%) and nonclassic (herpetiform, erythematous papulonodules, hyperkeratotic, eczematoid, zosteriform, and psoriasiform patterns: 17/70: 24.3%) (Figures 4(d)–4(i)). In contrast,* L. tropica* amastigotes were sized approximately 2-3 ± 0.23 *μ*m in length and 2.2 ± 0.11 *μ*m in width with amastigotes' shapes of round and/or oval within dry lesions (100%) (Figures 4(a), 4(b), and 4(j)). The grade of positive smears of* L. tropica* showed that all observed smears were +6.

Details of frequency of* Leishmania* lesions in suspected patients, lesion duration after adult sandflies' biting, lesion types, and lesions' numbers are shown in Table 1.

### 3.2. The Pixel Intensity of* Leishmania* by Targeting Three Genes of Cyt* b*, Microsatellite, and ITS-rDNA

**Figure 5:**
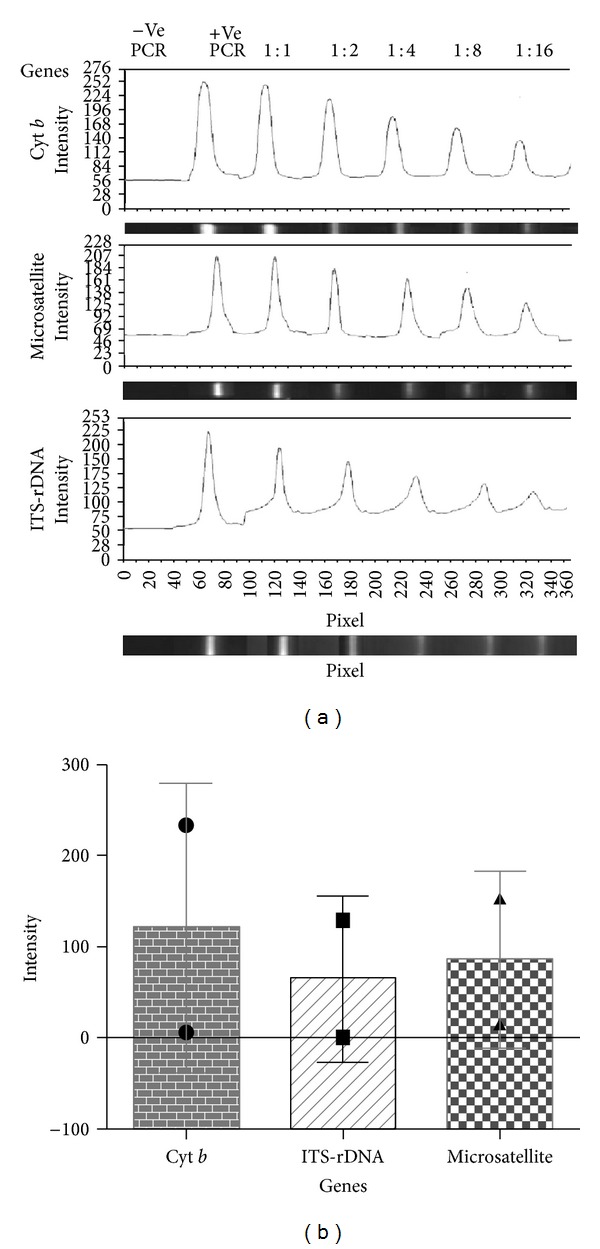
(a) Evaluating of three amplified PCR bands sensitivity based on provided serial dilution of target DNAs in ITS-rDNA, microsatellite, and Cyt *b* genes. Purified DNA (10 ng) from* L. major* was used as positive PCR control (+ve PCR). A negative PCR control (−ve PCR) without DNA was included in each reaction. Pixel intensity of PCR bands was calculated using Gel Analyser software (2010a) in different genes. (b) To assess the average of different dilutions of PCR bands based on three applied genes in this study, a graph presents the pixel intensity of Cyt* b*; 110, ITS-rDNA; 82 and ITS2-microsatellite; 90 (from left to right). Bar graph indicates the mean ± SEM.

The pixel intensity of applied genes was examined. The PCR products that were tested in gel electrophoresis rely on their chromatogram length. The sensitivity of PCR bands showed that the Cyt* b* gene has more pixel intensity (140) than microsatellite (125) and ITS-rDNA (110) genes in a minimum dilution, 1 : 16 (Figure 5(a)).

The average of different PCR dilutions was statistically analyzed and the mean of PCR bands demonstrated that Cyt* b* gene has more intensity (110) than microsatellite (90) and ITS-rDNA (82) (Figure 5(b)).

### 3.3. Identification of* Leishmania* Species by Targeting ITS-rDNA and Cyt* b* Genes Using RFLP

In this investigation,* L. major* and* L. tropica* were identified and typed molecularly in suspected patients in neglected foci of Khuzestan province. 139 out of 150 suspected patients were found infected with* Leishmania* parasites using three different genes of ITS-rDNA (90/139: 64.7%), Cyt* b* (30/139: 21.5%), and microsatellite (19/139: 13.6%) (Table 1).

37 out of 139 (26.6%) PCR products did not have enough DNA for digesting by* B*suRI (*H*aeIII) for ITS-rDNA and* S*sp1 for Cyt* b* (maxicircle) enzymes and/or sequencing of these genes. 102 positive samples were digested and some directly sequenced to identify* Leishmania* species. Of 139 suspected patients, 102 cases had 90* L. major* (64.7%), nine had* L. tropica* (6.47%), and three were unidentified (2.15%).

The digestion of* B*suRI (*H*aeIII) of ITS-rDNA and* S*sp1 of Cyt* b* enzymes for detection of different* Leishmania* species was performed by CLC DNA Workbench 5.2 software which are shown in Figure 2.

### 3.4. Sequencing and Phylogenetic Analyses of* Leishmania* Species by Targeting ITS-rDNA and Cyt* b* Genes

Only 25 out of 90* L. major* and all nine* L. tropica* were sequenced from both Cyt* b* and ITS-rDNA genes. All sequences of* Leishmania* parasites in this study were aligned, edited, analyzed, and compared with some* Leishmania* sequences of Cyt* b* and ITS-rDNA genes with those which had been previously submitted to the GenBank.* L. tropica* had more molecular variation than* L. major* (25* L. major* sequences versus nine* L. tropica*). Cyt* b* had a little more variation in comparison with ITS-rDNA gene for both* L. major* and* L. tropica*.

*L. major* had three haplotypes for Cyt* b* gene. One common haplotype was reported in 23* L. major* sequences which were similar with haplotype KHUZ57 (GenBank Accession no. AB095961). Two other haplotypes (haplotypes KHUZ17 and KHUZ19) were novel and unique (KM393221 and KM393217). For ITS-rDNA gene, all 25* L. (L.) major* sequences were similar (GenBank Accession nos. EF413075 and AJ300481) and no variation was found.

Of our nine* L. tropica* sequences, three haplotypes were identified distinctively including a common and two new haplotypes for Cyt* b* gene. The common haplotype included six* L. tropica* sequences which were similar with haplotype KHUZ22 (GenBank Accession no. EF579904). Two new haplotypes were seen in three remained sequences. Haplotype KHUZ02 had two similar sequences (KM393218) and the other one (haplotype KHUZ05) was unique (KM393219). For ITS-rDNA gene of* L. tropica,* two haplotypes were found. One common haplotype KHUZ11 had eight similar sequences (GenBank Access no. FN677343) and one new haplotype KHUZ07 which was unique (KM393220). Three* Leishmania* parasites were unidentified because of not enough DNA for direct sequencing or unreadable sequences and/or mixed infections (Table 1).

A coinfection was found from a man (age about 24), resident of Kotabdullah village (Ahvaz region) who had* L. major* in left hand,* L. tropica* in face, and a* Crithidia* in the lesion of right hand. This could be very interesting to notice when different parasites are existed in one patient; therefore, each lesion should be identified to species and examined separately.

**Figure 6:**
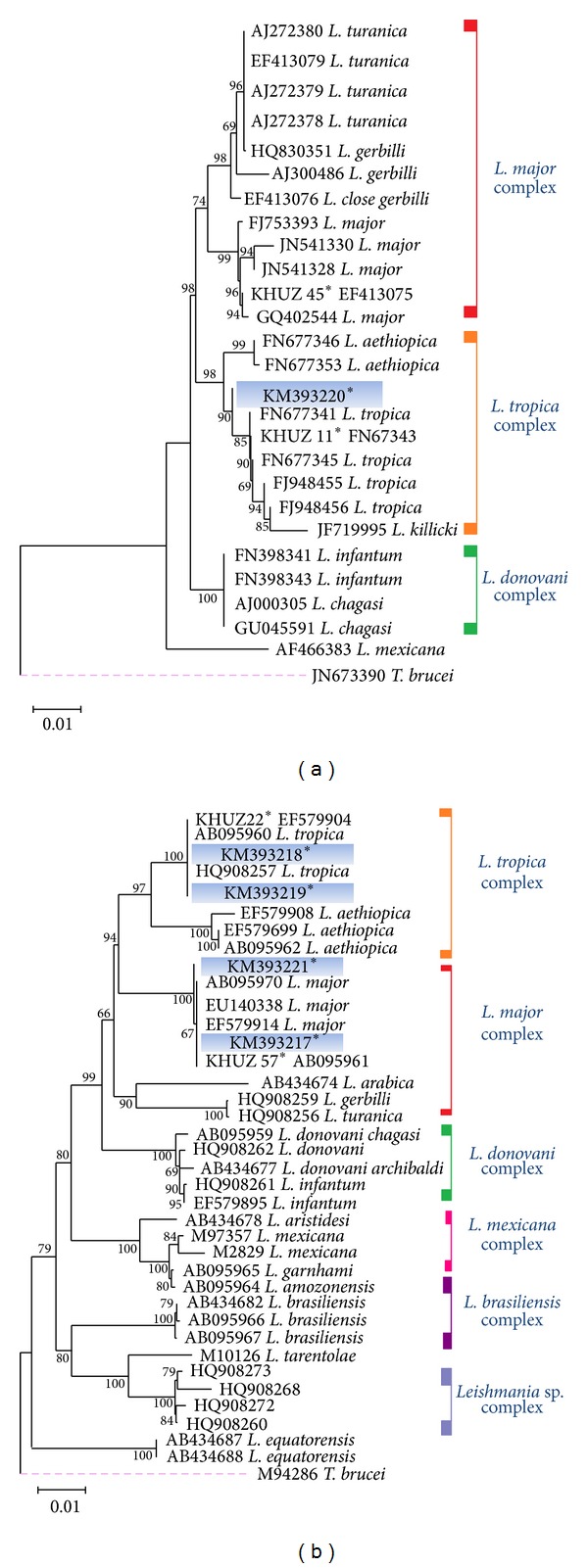
The Phylogeny of* Leishmania* species according to the maximum-likelihood (ML), tree was conducted based on the multiple sequence alignment (haplotypes) of ITS-rDNA (a) and Cyt* b* (b) genes by MEGA5.05. Only bootstrap values of higher than 70% are indicated on each branch. Distance represents the number of base substitutions per site.* Trypanosoma brucei* (M94286; Cyt *b* and JN673390; ITS-rDNA) is the outgroup branch. ∗ = Analyzed sequences in this study are shown in (a) and (b) for* L. major* and* L. tropica* complexes.

Phylogenetic analyses were conducted using our new and common haplotypes of both genes and* Leishmania* species with those which were submitted in GenBank using MEGA v5.05.* Trypanosoma brucei* was considered as an out group branch (GenBank Accession nos. M94286 and JN673390) (Figures 6(a) and 6(b)).

## 4. Discussion

In this investigation, two* Leishmania* species (*L. major* and* L. tropica*) were unequivocally identified in suspected patients of the three different districts including Sosangerd, Dashte Azadegan, and Hoveyze in western bordering with Iraq, Ahvaz as capital city of Khuzestan province in central and also Ramhormoz and Ramshir in eastern Khuzestan province where all these three different regions are not considered widely as ZCL foci (Figure 1) [4]. In north of Khuzestan province, Shush is well known as an endemic focus of ZCL [44]. In our study, prevalence of ZCL in ignored locations of disease was unexpectedly high (17.1 per 10,000) to be compared with previous report, 8 per 10,000 [4]. Also,* L. major* and* L. tropica* were found in Sosangerd, Dashte Azadegan, Hoveyze, and Ahvaz, while* L. major* was only found in Ramhormoz and Ramshir. This maybe because of sandflies' species or reservoir hosts distribution in different regions.* Phlebotomus papatasi* and* P.* (*Paraphlebotomus*)* sergenti* were collected and identified in these two first locations (Sosangerd, Dashte Azadegan, and Ahvaz). Those are the vectors which circulate in these areas but only* P. papatasi* was found in the third region (Ramhormoz and Ramshir) whilst the rodent* Tatera indica* and humans/dogs are reservoir hosts of* L. major* and* L. tropica,* respectively in the region [45, 46].

In this study, three different genes were employed to detect* Leishmania* species. The advantages of RFLP for Cyt *b* gene were sharp PCR bands with high sensitivity in the shortest possible time (5 min., rapid digestion) compared with ITS-rDNA and microsatellite (Figures 3(a), 3(b), 5(a), and 5(b)). These could depend on high multicopy sequences of Cyt *b* per cell (comprised of 20–50 maxicircles, 20–35 kb in size) and presence of an unusually long noncoding region (5–20 kb), including five nonedited MURF1, 12S, COI, ND1, and 9S genes [32].

Regular and different irregular shapes of amastigotes of* L. major* were found in various clinical patterns, this can be stated by various reservoir hosts and vectors compared to* L. tropica* in the region. Only regular shape of amastigotes was identified in* L. tropica*, in contrast, more molecular variation was observed in* L. tropica* sequences. This could be justified by the genetic characters of this parasite, because GC content of* L. tropica* is lower than* L. major* [10, 47, 48].

Unidentified* Leishmania* species could be inducted by mixed pathogens or non-pathogens with* L. major, L. tropica,* and/or* Crithidia* [49–51]. Unidentified and mixed infections could be resolved by cloning of genes which was not the purpose of our approved research project [52].

A coinfection of* L. major, L. tropica,* and* Crithidia* in different sites on body of the man in our research from Khuzestan province can approve the existence of these pathogens and nonpathogens in suspected patents, but the surprising matter is that how nonpathogen of* Crithidia* made acute lesion in right hand of patient. More remarkable finding of co-infection were wet lesion for both* L. tropica* and* Crithidia* which expecting only for* L. major* ulcer.

*L. major* was isolated and clearly identified in three clinical forms (wet, dry, and mixed ulcers), while we expected to observe only in wet lesions. These unexpected observations of three different ulcers should be described by the ability of* L. major* isolates to have tropism to clinical patterns isolated in southwestern Iran [18, 53].

No correlation was found between the phenotypic character (amastigote morphometrics) and genotypic features in* L. major* (*P* > 0.05) using ITS-rDNA (nucleus) and Cyt* b* (mitochondrial) and microsatellite in the most neglected regions of the middle Khuzestan, Iran. Because different amastigotes' shapes of* L. major* had the same sequences and no molecular variation was detected. The amastigote shape of* L. tropica* was observed only in regular shape but they had more molecular variations based on mentioned genes. High GC content of* L. major* (52.5%) is more stable than low GC content of* L. tropica* and it should be considered because of triple hydrogen bonds of the GC pair and mainly due to stacking interaction [10, 47, 48, 54].

More variation and heterogeneity of* L. tropica* in our study also could explain the same as ITS-1 ribosomal DNA sequence of* Echinococcus* which showed there are two turnover mechanisms, namely, unequal crossing over/transposition and slippage [55].

These findings attenuate explicitly relationship between genotypic and phenotypic characters of CL in* L. major* and* L. tropica*, which potentially indicates that genetic variation aspects are not always able to influence the formation of morphometric features or even different clinical appearances [8, 9, 56, 57].

A comprehensive analysis on limits of morphological diversity in any protozoan parasite showed that morphogenetic constraints and extrinsic selection pressures are associated with the full diversity of trypanosomatid morphology including juxtaform and liberform superclasses [19].

Several factors can separately affect the clinical features of* L. major* lesions. These are including immunosuppressive diseases, migration of nonindigenous populations, host factors, the number of the inoculated parasites, using oral steroids, the site of inoculation, and even wound contamination with inorganic ingredients [8, 9]. Considering challenge on phenotypic and genotypic patterns of* L. tropica* in suspected patients in Khuzestan was that* L. tropica* had no polymorphic amastigotes but have more molecular variations with many differentiations in nucleotides of each sequences (haplotype) [10, 17].

Some studies revealed molecular diversity in mini-exon gene of CL agents (*L. major* and* L. tropica*) in Khuzestan isolates but the heterogeneity was not coordinated with the size of lesions [22, 23].

Most publications showed that causative agent of cutaneous leishmaniasis is* L. major;* however Maraghi et al. (2007) identified both* L. major* (90%) and* L. tropica* (10%) in this regions [12, 44, 58].

Hajjaran et al. (2013) using only ITS-rDNA gene showed* L. tropica* with 3.6% polymorphisms and* L. major* with 7.3% genetic variations in Iran [59], whilst we found more variations in* L. tropica* to be compared with* L. major* using Cyt* b* mitochondrial and ITS-rDNA nucleus genes which are able to draw a real conception of genetic diversity among* Leishmania *species.

Many studies were carrying out regarding identifications of* Leishmania* parasites in human, reservoirs hosts, and vectors in Iran and the world which some of them correlated the molecular variations and heterogeneity of* Leishmania* species with clinical signs [59–62].

## 5. Conclusions

In this study,* L. major* and* L. tropica* were firmly identified in suspected patients with a coinfection of* L. major*,* L. tropica,* and* Crithidia* and some unidentified* Leishmania* which could be mixed of these three or with the expectation of the existence of* L. turanica* which recently was reported from human in north of Iran [7]. It is crucial that simultaneous screening of both valuable mitochondrial (Cyt* b*) and nucleus (ITS-rDNA) genes with morphometric, molecular, and phylogenetic analyses can provide an appropriate approach and reliable methods to identify* Leishmania* haplotypes, strains, and species.

## Figures and Tables

**Table 1:** Characteristics of confirmed patients to cutaneous leishmaniasis based on collection study site, personal characters, microscopic observation, and molecular methods.

Province regions (positions)	Collection site	Characteristics																		Microscopic observation								Molecular methods						
	Cities and villages	Sex		Age groups (years)							Lesion duration (month)			Lesion number			Lesion types			*Leishmania* amastigotes' positive grading numbers: 1+ to 6+								*Leishmania* amplification by targeted genes				RFLP with *B*suRI & *S*spI, sequencing and phylogenetic analyses		
		Female	Male	<1	1–3	3–5	5–10	10–15	15–25	>25	<1	2-3	>3	Single	Double	Multiple	Wet	Dry	Mixed	1+	2+	3+	4+	5+	6+	Total		ITS-rDNA/ & Cyt *b* & ITS-microsatellite +ve				*L.* *major* +ve	*L.* *tropica* +ve	Unidentified; *Leishmania* sp
																										−ve	+ve	ITS-rDNA	Cyt *b *	∗Mic	−ve			
Khuzestan																																		
Ahvaz (central)	Kotabdullah	4	∗∗1	0	1	1	1	2	0	0	2	2	1	3	1	1	4	1	0	0	1	0	2	1	1	0	5	3	1	1	0	2	2	1
	Damane kochak	4	6	0	1	2	1	1	4	1	7	1	2	6	2	2	7	2	1	1	1	1	4	2	1	3	10	8	0	2	3	5	0	0
	Ashari bozorg	3	7	0	1	1	3	0	3	2	3	4	3	2	5	3	7	2	1	1	1	0	2	2	4	1	10	7	1	0	1	6	0	0
	Bahonar	2	2	0	0	0	0	2	1	1	3	1	0	0	0	4	4	0	0	0	0	0	1	1	1	1	4	3	1	0	1	3	1	0
	Sheyban	2	3	0	0	0	2	0	2	1	3	2	0	2	2	1	1	4	0	2	1	1	0	1	0	0	5	4	1	0	0	5	0	0
	Koye abozar	0	2	1	0	0	1	0	0	0	1	1	0	0	0	2	1	0	1	0	0	0	2	0	0	0	2	1	1	0	0	2	0	0
	Kian abad	0	5	0	0	0	2	0	3	0	2	2	1	5	0	0	3	2	0	0	1	1	1	2	0	0	5	3	2	0	0	2	0	0
	Shahrake bargh	2	6	0	1	1	2	0	2	2	6	0	2	4	3	1	7	0	1	1	2	1	1	0	3	1	8	3	2	2	1	3	1	0
	Padad	2	3	0	0	1	2	0	0	2	4	1	0	4	1	0	2	2	1	0	0	4	1	0	0	0	5	4	1	0	0	3	0	0
	Sadeghiyeh	2	0	0	0	0	0	0	0	2	2	0	0	2	0	0	1	0	1	0	0	0	2	0	0	1	2	2	0	0	0	2	0	0
	Malashiye	0	3	0	0	0	1	0	2	0	2	1	0	2	1	0	1	2	0	0	1	1	1	0	0	0	3	3	0	0	0	3	0	0
	Amaniyeh	1	2	2	1	0	0	0	0	2	2	0	1	2	1	0	2	1	0	0	0	1	2	0	0	0	3	3	1	1	0	2	0	0
	400 dastghah	1	3	0	0	2	0	2	0	0	3	0	1	1	3	0	1	3	0	0	0	2	0	2	0	0	4	2	2	0	0	3	0	0
	Koye bonovat	1	2	0	0	1	0	0	0	2	2	1	0	2	0	1	1	2	0	0	0	0	3	0	0	1	3	2	1	0	0	2	1	0
Dashteazadegan (western)	Hajiyeh	1	2	0	0	0	1	0	1	1	0	2	1	1	2	0	3	0	0	0	0	1	1	0	1	0	3	2	1	0	0	2	0	1
	Soydani	0	4	0	1	0	1	2	0	0	2	1	1	1	1	2	3	1	0	0	0	1	0	1	1	1	4	3	2	0	1	3	1	0
	Bostan centre	3	3	0	0	0	0	2	2	2	2	3	1	0	3	3	2	3	1	1	2	1	1	1	0	0	6	5	1	4	0	3	0	0
	Shakeriye	2	3	0	0	0	1	2	2	0	2	2	1	2	3	0	3	1	1	0	0	2	1	0	2	0	5	3	2	0	0	4	0	0
	Jalaliye	1	2	1	1	0	1	0	0	0	0	1	2	0	2	1	2	1	0	0	1	0	0	0	1	1	3	4	1	1	1	2	1	0
	Albo afri	1	3	1	1	2	0	0	0	0	4	0	0	0	1	3	3	0	1	0	0	0	1	2	1	0	4	2	2	0	0	4	0	0
	Malekiyeh	0	2	0	0	0	0	1	0	1	0	2	0	1	1	0	1	1	0	0	0	1	0	0	0	1	2	1	0	1	1	2	0	0
	Koye abodi	0	2	0	0	0	0	1	1	0	0	2	0	1	0	1	2	0	0	0	0	0	1	0	1	0	2	2	0	0	0	2	0	0
	Hoveyze	3	7	1	0	1	1	0	3	4	5	2	3	1	4	5	5	5	0	1	2	2	2	1	2	0	10	3	2	1	0	5	1	1
	Farhangian	1	6	0	1	1	1	1	3	0	2	5	0	1	5	1	5	1	1	0	4	1	2	0	0	1	7	3	1	1	1	5	1	0
Ramhormoz (eastern)	Darre khosh	3	6	1	0	0	0	2	5	1	8	1	0	3	2	4	7	2	0	0	1	0	5	1	2	0	9	5	1	2	0	7	0	0
	Shardin	1	4	1	1	0	0	0	1	2	0	2	3	2	2	1	3	1	1	0	1	0	1	3	0	0	5	4	1	1	0	3	0	0
	Shamilan	3	3	1	2	0	0	1	2	0	3	2	1	1	2	3	5	1	0	0	1	1	1	1	1	1	6	2	1	1	1	5	0	0
	Total	**43**	**94**	**9**	**12**	**13**	**21**	**19**	**37**	**26**	**66**	**47**	**24**	**51**	**47**	**39**	**86**	**38**	**11**	**7**	**20**	**22**	**45**	**21**	**22**	**13**	**137**	**90**	**30**	**19**	**11**	**90**	**9**	**3**
		**137**		**137**							**137**			**137**			**137**			**137**						**150**		**139**				** 102**		

* = microsatellite, ∗∗1 = One patient had coinfections with *L. major*, *L. tropica,* and *Crithidia*.

**Table 2:** Personal information, lesion characteristics, shapes, and sizes of amastigotes in *L. major* and *L. tropica* isolated from patients in three locations of study sites.

Criteria		Lesion type in *L. tropica *	Lesion types in *L. major *				Morphometric characteristics of *L*. *tropica* amastigotes			Morphometric characteristics of *L*. *major *amastigotes						
							Size of amastigotes (*µ*m)	Shapes of amastigotes		Sizes of amastigotes (*µ*m)		Shapes of amastigotes				
								Regular				Regular		Irregular		
Age group (years)		Dry	Wet		Dry	Mixed	2-3	Round	Oval	3-4	>4	Round	Oval	Pear	Cigarette	Spindle
Age ranges	Total		∗C	∗NC												
<1	4	0	2	2	0	0	0	0	0	4	0	1	2	0	1	0
1–3	9	1	5	2	1	0	1	1	0	6	2	2	3	2	1	0
3–5	11	1	5	1	2	1	1	1	0	7	3	1	9	0	0	0
5–10	12	2	5	2	3	1	2	1	1	7	3	2	6	1	1	0
10–15	18	2	10	3	2	1	2	1	1	12	4	3	11	1	1	0
15–25	27	2	17	3	3	2	2	1	1	20	5	2	20	1	1	1
>25	18	1	12	4	1	0	1	0	1	15	2	1	11	1	2	2

Total (%)	99	9	56	17	12	5	9	5 (55.5)	4 (44.4)	71 (78.9)	19 (21.1)	12 (13.3)	62 (68.9)	6 (6.7)	7 (7.7)	3 (3.3)
			70 (77.7)		17 (18.9)							74 (85.5)		16 (17.7)	
			90					9		90		90				

*C: Classic and ∗NC: Nonclassic.
